# Trop-2 in Upper Tract Urothelial Carcinoma

**DOI:** 10.3390/curroncol29060312

**Published:** 2022-05-30

**Authors:** Eisuke Tomiyama, Kazutoshi Fujita, Kosuke Nakano, Ken Kuwahara, Takafumi Minami, Taigo Kato, Koji Hatano, Atsunari Kawashima, Motohide Uemura, Tetsuya Takao, Hiroaki Fushimi, Kotoe Katayama, Seiya Imoto, Kazuhiro Yoshimura, Ryoichi Imamura, Hirotsugu Uemura, Norio Nonomura

**Affiliations:** 1 Department of Urology, Osaka University Graduate School of Medicine, Osaka 565-0871, Japan; tomiyama@uro.med.osaka-u.ac.jp (E.T.); kosukenakano97@gmail.com (K.N.); kato@uro.med.osaka-u.ac.jp (T.K.); hatano@uro.med.osaka-u.ac.jp (K.H.); kawashima@uro.med.osaka-u.ac.jp (A.K.); uemura@uro.med.osaka-u.ac.jp (M.U.); imamura@uro.med.osaka-u.ac.jp (R.I.); nono@uro.med.osaka-u.ac.jp (N.N.); 2 Department of Urology, Kindai University Faculty of Medicine, Osaka 589-8511, Japan; ken_830709@yahoo.co.jp (K.K.); minami@med.kindai.ac.jp (T.M.); yoshimur@med.kindai.ac.jp (K.Y.); huemura@med.kindai.ac.jp (H.U.); 3 Department of Urology, Osaka General Medical Center, Osaka 558-8558, Japan; takao@gh.opho.jp; 4 Department of Pathology, Osaka General Medical Center, Osaka 558-8558, Japan; hiroaki-fushimi@gh.opho.jp; 5 Laboratory of Sequence Analysis, Human Genome Center, The Institute of Medical Science, The University of Tokyo, Tokyo 108-8639, Japan; k-kataya@hgc.jp (K.K.); imoto@hgc.jp (S.I.); 6 Division of Health Medical Intelligence, Human Genome Center, The Institute of Medical Science, The University of Tokyo, Tokyo 108-8639, Japan

**Keywords:** trophoblast cell surface antigen 2, *TACSTD2*, upper tract urothelial carcinoma, immunohistochemical analysis, RNA sequencing, sacituzumab govitecan

## Abstract

Trophoblast cell surface antigen 2 (Trop-2, encoded by *TACSTD2*) is the target protein of sacituzumab govitecan, a novel antibody-drug conjugate for locally advanced or metastatic urothelial carcinoma. However, the expression status of Trop-2 in upper tract urothelial carcinoma (UTUC) remains unclear. We performed immunohistochemical analysis of 99 UTUC samples to evaluate the expression status of Trop-2 in patients with UTUC and analyze its association with clinical outcomes. Trop-2 was positive in 94 of the 99 UTUC samples, and high Trop-2 expression was associated with favorable progression-free survival (PFS) and cancer-specific survival (*p* = 0.0011, 0.0046). Multivariate analysis identified high Trop-2 expression as an independent predictor of favorable PFS (all cases, *p* = 0.045; high-risk group (pT3≤ or presence of lymphovascular invasion or lymph node metastasis), *p* = 0.014). Gene expression analysis using RNA sequencing data from 72 UTUC samples demonstrated the association between high *TACSTD2* expression and favorable PFS (all cases, *p* = 0.069; high-risk group, *p* = 0.029). In conclusion, we demonstrated that Trop-2 is widely expressed in UTUC. Although high Trop-2 expression was a favorable prognostic factor in UTUC, its widespread expression suggests that sacituzumab govitecan may be effective for a wide range of UTUC.

## 1. Introduction

Upper tract urothelial carcinoma (UTUC) is a relatively rare disease, accounting for 5–10% of all urothelial carcinomas (UC) [1,2]. Patients with UTUC often present with invasive or metastatic disease at diagnosis and, therefore, have a poorer prognosis than those with urothelial bladder cancer (UBC) [1]. Phenotypic and genetic differences between both malignancies have also been found, and increasing evidence suggests that UTUC could be a distinct disease from UBC [2]. Thus, it is important to develop optimal management strategies for UTUC.

Locally advanced or metastatic UC, including UTUC, is an incurable disease with poor survival. Until recently, patients with metastatic UC have had limited treatment options and exhibit tumor progression after platinum chemotherapy, immune checkpoint inhibitor treatment, or both [3,4]. However, the therapeutic potential for metastatic UC was expanded by the accelerated approval of enfortumab vedotin, an antibody–drug conjugate (ADC) targeting Nectin-4, by the US Food and Drug Administration (FDA) in 2019 [5]. In April 2021, sacituzumab govitecan, an ADC targeting trophoblast cell surface antigen 2 (Trop-2), became a treatment option for patients with locally advanced or metastatic UC who previously received a platinum-containing chemotherapy and a programmed cell death 1 (PD-1) or PD-1 ligand 1 (PD-L1) inhibitor [6]. Sacituzumab govitecan, which is an anti-Trop-2 monoclonal antibody conjugated to SN-38, is an active metabolite of irinotecan that creates breaks in double-stranded DNA and leads to apoptosis [7]. Previous studies have indicated that cells overexpressing Trop-2 are highly sensitive to sacituzumab govitecan [8,9,10,11]. Therefore, it is clinically important to evaluate the expression of Trop-2 in solid tumors to predict the efficacy of sacituzumab govitecan.

Trop-2 is a 40-kDa transmembrane glycoprotein encoded by the single-exon gene *TACSTD2* and is highly expressed on the surface of various epithelial cancer cells, including UBC [10,11,12,13,14,15,16,17,18,19]. However, the expression status of Trop-2 and its prognostic significance in UTUC have not been fully investigated. Therefore, in this study, we investigated the expression pattern of Trop-2 using tissue microarray (TMA) specimens of 99 patients with UTUCs. Furthermore, we analyzed the association of Trop-2 expression with clinical outcomes in UTUC. We also performed gene expression analysis using the RNA-sequencing data from 72 UTUC samples and compared clinical outcomes between the high and low *TACSTD2* expression groups.

## 2. Materials and Methods

### 2.1. Patients and Tissue Samples

For the immunohistochemical analysis, the UTUC-TMA was constructed using spotted triplicate UTUC samples from dominant tumors/invasive components, if present, of 99 patients with non-metastatic UTUC. These patients underwent radical nephroureterectomies performed at Osaka General Medical Center between 1997 and 2011, as described previously [20,21,22,23,24,25].

For the gene expression analysis, 72 patients with non-metastatic UTUC who underwent radical nephroureterectomy at Osaka University Hospital between 2016 and 2020 were enrolled. Surgically resected UTUC samples were immersed in RNAlater tissue storage reagent (Thermo Fisher Scientific, Waltham, MA, USA) and stored at −20 °C.

Ethical approval for the study was obtained from each local institutional review board (IRB), including: Osaka General Medical Center Institutional Review Board (IRB), Protocol Number 25–2014 and Osaka University Hospital IRB, Protocol Number #13397-14. Written informed consent was obtained from all patients before recruitment. Tumor progression was defined as the development of non-lower urinary tract lesions, including recurrence at the site of nephroureterectomy and lymph node or visceral metastasis. The high-risk group consisted of patients who had a pathologic stage of ≥pT3 or positive lymphatic invasion or lymph node metastasis [26].

### 2.2. Immunohistochemical Analysis

Immunohistochemical staining for Trop-2 was performed using 4 μm-thick paraffin-embedded tissue sections from the UTUC-TMA, which were deparaffinized using xylene and a graded series of ethanol concentrations. For Trop-2 antigen retrieval, the sections were treated with Tris-ethylenediaminetetraacetic acid (EDTA) buffer (pH 7.0) and steamed by placing them above boiling water for 20 min. Endogenous peroxidase activity was blocked by incubating the sections with 0.3% hydrogen peroxide for 5 min, followed by overnight incubation with primary antibodies against Trop-2 (1:200; SC-376181, Santa Cruz Biotechnology, Dallas, TX, USA) at 4 °C.

Then, we used the EnVision + system-horseradish peroxidase (HRP)-labeled polymer anti-mouse (DAKO) according to the manufacturer’s instructions. Sections were counterstained with hematoxylin, dehydrated using a graded series of ethanol concentrations, cleared in xylene, and mounted for viewing under a microscope. The intensity and extent of Trop-2 expression were determined using the histochemical scoring system (H-score), which was based on the product of the staining intensity (score, 0–3+) and percentage of stained cells (0–100%) at a given intensity. Specimens were subsequently classified as negative (0+, H-score 0–14), low (1+, H-score 15–99), moderate (2+, H-score 100–199), and high (3+; H-score, 200–300).

### 2.3. TACSTD2 Gene Expression Analysis in UTUC

RNA sequencing analysis was performed as previously reported [27]. Briefly, total RNA was isolated from 73 UTUC tumor samples using the RNeasy mini kit (QIAGEN, Venlo, Netherlands) according to the manufacturer’s protocol. The RNA integrity was verified using an Agilent 2100 bioanalyzer with RNA nano reagents (Agilent Technologies, La Jolla, CA, USA), and RNA was subjected to polyA+ selection and chemical fragmentation. The 100-bp RNA fraction was used to construct cDNA libraries using the TruSeq Stranded mRNA Prep kit (Illumina, San Diego, CA, USA) and the obtained paired-end libraries were sequenced using the Illumina NovaSeq6000 platform with a standard 100-bp paired-end read protocol at Macrogen Japan. *TACSTD2* gene expression values were estimated from the RNA sequencing data using the Genomon pipeline (https://github.com/Genomon-Project/genomon-docs/tree/v2.0 (accessed on 4 January 2022). The alignment was performed with the STAR aligner (v.2.5.2a) against the hg19 human genome. BAM files named Aligned.sortedByCoord.out.bam, generated using the STAR software, were used to quantify the expression data using GenomonExpression. Patients were divided into high and low *TACSTD2* expression groups (*n* = 36 each) based on the median value to evaluate the association between *TACSTD2* expression and prognosis.

### 2.4. Statistical Analyses

Statistical analyses were performed using JMP Pro 16.0.0 (SAS Institute Inc., Cary, NC, USA) and data were visualized using GraphPad Prism version 7.05 (GraphPad Software, San Diego, CA, USA). Fisher’s exact test was used to evaluate the association between categorized variables. The survival rates were determined using the Kaplan–Meier method, whereas the log-rank test was used for comparison between groups. The Cox proportional hazards model was used to determine the statistical significance of prognostic indicators in univariate and multivariate settings. *p*-values < 0.05 were considered statistically significant and *p*-values < 0.1 were considered statistically trending.

## 3. Results

### 3.1. Patient Characteristics

The clinicopathological characteristics and outcomes of the 99 and 72 patients (using immunohistochemical and RNA sequencing analyses, respectively) are summarized in Table 1. Sixty of 99 patients (60.6%) and 33 of 72 patients (45.8%) were classified in the high-risk group. None of the patients received neoadjuvant therapy before tissue collection; however, 26 of 99 patients (26.3%) and 12 of 72 patients (16.7%) underwent adjuvant chemotherapy. Tumor extension was observed in 38 of 99 patients (38.4%) and 25 of 72 patients (34.7%).

### 3.2. Trop-2 Expression in UTUC and Association with Clinicopathological Characteristics

**Figure 1:**
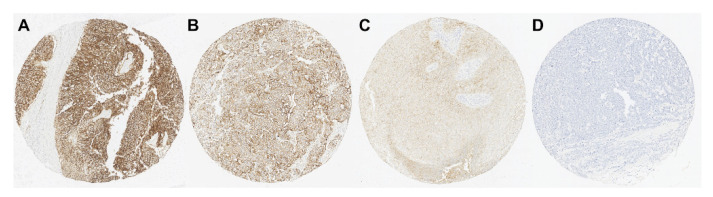
Immunohistochemical expression of trophoblast cell surface antigen 2 (Trop-2) in upper tract urothelial carcinoma (UTUC) tissue microarray specimens. UTUC tissue with (**A**) high (3+), (**B**) moderate (2+), (**C**) low (1+), and (**D**) negative (0+) expression.

Typical patterns of Trop-2 immunohistochemical expression in UTUC-TMA specimens are portrayed in Figure 1. Trop-2 positivity was detected in 94 (94.9%; 25 [25.3%] low, 42 [42.4%] moderate, 27 [27.3%] high) of the 99 UTUC samples. Next, our analysis of the association of Trop-2 expression with the clinicopathological profiles of 99 patients with UTUC (Table 2) indicated no association with patient sex, tumor location, tumor grade, lymphovascular invasion, or lymph node metastasis (0 vs. 1+/2+/3+; 0/1+ vs. 2+/3+; 0/1+/2+ vs. 3+). Moreover, high Trop-2 expression was detected at a significantly higher level in non-muscle-invasive (40.5%) than in muscle-invasive (19.4%) tumors (*p* = 0.020).

### 3.3. Immunohistochemical Analysis of Associations of Trop-2 Expression with Patient Outcomes

**Figure 2:**
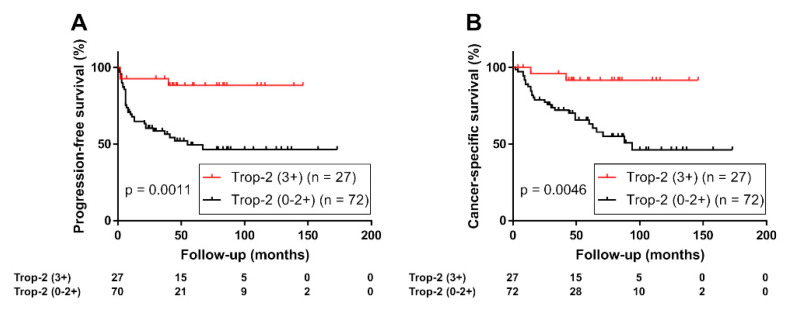
(**A**) Progression-free survival and (**B**) cancer-specific survival of 99 patients with upper tract urothelial carcinoma stratified using expression of trophoblast cell surface antigen 2 (Trop-2, 0/1+/2+ vs. 3+).

**Figure 3:**
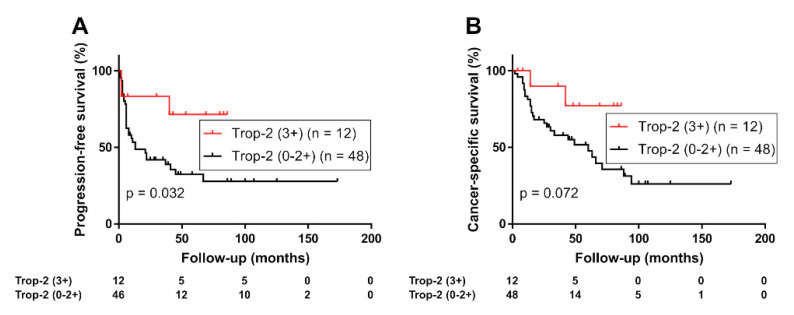
(**A**) Progression-free survival and (**B**) cancer-specific survival of 60 patients with high-risk upper tract urothelial carcinoma (with a pathologic stage of ≥pT3 or positive lymphatic invasion or lymph node metastasis) stratified using expression of Trop-2 (0/1+/2+ vs. 3+).

The Kaplan–Meier analysis of the prognostic value of Trop-2 expression status in UTUC revealed that patients with high Trop-2 expression (*n* = 27) had a significantly lower risk of tumor progression (Log-rank test, *p* = 0.0011; Figure 2A) or cancer-specific mortality (Log-rank test, *p* = 0.0046; Figure 2B) than those with moderate, low, or negative Trop-2 tumor expression (*n* = 72). In a sub-group of patients with muscle-invasive tumors (*n* = 62), high Trop-2 expression was also associated with favorable PFS (*p* = 0.032, Supplementary Figure S1A) and cancer-specific survival (*p* = 0.061, Supplementary Figure S1B). In addition, although limited to the high-risk group (*n* = 60), highTrop-2 expression was associated with favorable PFS (*p* = 0.032, Figure 3A) and cancer-specific survival (*p* = 0.072, Figure 3B).

Next, the multivariate analysis with the Cox proportional hazard model was performed to determine whether highTrop-2 expression was an independent prognostic factor in patients with UTUC. The results demonstrated that highTrop-2 expression was an independent predictor of favorable PFS in UTUC (all cases: hazard ratio [HR], 0.29; 95% confidence interval [CI], 0.087–0.97; *p* = 0.045]; high-risk group: HR, 0.27 [95% CI, 0.081–0.88; *p* = 0.031, Table 3 and Table 4).

### 3.4. Associations of TACSTD2 Gene Expression with Patient Outcomes

**Figure 4:**
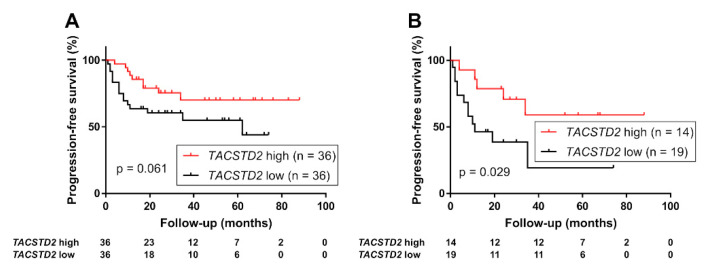
Progression-free survival in (**A**) 72 patients with upper tract urothelial carcinoma (UTUC) and (**B**) 33 patients with high-risk UTUC (with a pathologic stage of ≥pT3 or positive lymphatic invasion or lymph node metastasis) stratified using expression of *TACSTD2* gene (high vs. low).

Finally, we evaluated the association between *TACSTD2* expression and prognosis using the RNA sequencing data in a different patient cohort (*n* = 72). The Kaplan–Meier analysis demonstrated that the group with high *TACSTD2* expression had a more favorable PFS than the low expression group (Log-rank test, all cases, *p* = 0.061; high-risk group, *p* = 0.029, Figure 4). In the multivariate analysis with Cox proportional hazard model, high *TACSTD2* expression was identified as an independent predictor of favorable PFS in high-risk UTUC (HR, 0.21; 95% CI, 0.057–0.75; *p* = 0.017, Supplementary Table S1).

## 4. Discussion

In this study, we demonstrated that Trop-2, the target protein of sacituzumab govitecan, was widely expressed in UTUC and that its high expression was associated with a better prognosis. To date, it has been reported that Trop-2 is highly expressed in various types of solid tumors, such as ovarian [10], cervical [11,12], colorectal [13,14], gastric [15], pancreatic [16], and breast cancers [17]. Sacituzumab govitecan was approved by FDA for patients with metastatic triple negative breast cancer who received at least two prior therapies in April 2021 [28]. Additionally, elevated expression of Trop-2 has also been reported in UC [18,19]. The TROPHY-U-01 phase II trial evaluated the efficacy of sacituzumab govitecan in locally advanced or metastatic UC and found an objective response rate (ORR) of 27% (31 of 113 patients) and a decrease in detectable disease in 77% of the patients [29]. Thus, sacituzumab govitecan has become a novel therapeutic option for metastatic UC after prior platinum-based therapies and immune checkpoint inhibitors [6]. However, previous studies have focused only on UBC; therefore, the expression of Trop-2 in UTUC has not been fully investigated. UTUC has been suggested to be a distinct disease from UBC [2]; as such, assessing the expression of proteins in UTUC is important even if their expression in UBC is well understood. Indeed, we previously indicated that Nectin-4, the target protein of enfortumab vedotin, is expressed at lower levels in UTUC than in UBC [25]. This finding supports the results of a subgroup analysis in a phase 3 trial, which indicated that enfortumab vedotin was less effective in UTUC than in UBC [30]. Our present study indicated that Trop-2 was highly expressed in UTUC and UBC, where it was reported to be 83% [31]. This finding suggests that sacituzumab govitecan may be a treatment option for advanced UTUC in addition to UBC.

Additionally, we revealed that the impact of Trop-2 on cancer prognosis may differ between UBC and UTUC. Overexpression of Trop-2 has been associated with increased tumor aggressiveness and poor prognosis in several types of cancers, including UBC [12,13,14,15,16,17,18,19,32]. Furthermore, Avellini et al. [18] reported that Trop-2 expression increases with increasing severity of UBC, and Trop-2 enhances proliferation and migration of UBC in vitro. Zhang et al. [19] found high expression of Trop-2 was associated with shorter recurrence-free survival in non-muscle-invasive UBC patients. However, in the present study, we revealed that high Trop-2 expression was associated with a good prognosis in UTUC. This finding is inconsistent with that reported in UBC; therefore, in addition to the immunohistochemical analysis, we also evaluated the gene expression of another cohort. The results of the analysis indicated that the high *TASCSTD2* expression group still indicated a favorable prognosis. The loss of Trop-2 has been reported to promote carcinogenesis and features of epithelial to mesenchymal transition in specific cancers such as head and neck squamous cell carcinoma [33]. Consequently, the association between high Trop-2 expression and favorable prognosis in UTUC might be a unique feature of UTUC that differs from what occurs with UBC. Further studies are expected to improve knowledge of the differences between UTUC and UBC [34].

The present study has some limitations that are worth mentioning. First, the UTUC specimens analyzed were obtained using radical nephroureterectomy and included samples from low-risk UTUC patients as well. Metastatic UTUC possesses a more aggressive phenotype than the localized form and may exhibit differential marker expression. Second, immunohistochemical analysis is a semi-quantitative evaluation method, thus, the staining pattern may not represent the actual expression level of the protein. Third, considering that the response rate of patients with advanced bladder cancer to sacituzumab govitecan was limited to 27% in the TROPHY-U-01 trial [29], the expression of Trop-2 does not ensure the efficacy of sacituzumab govitecan. An initial small pilot study (IMMU-132) suggested that high Trop-2 expression in UC was positively correlated with treatment response [31]. However, the relationship between Trop-2 expression pattern and the actual patient response rate to sacituzumab govitecan must be confirmed in future clinical trials.

In conclusion, our study indicated that Trop-2 is widely expressed in UTUC. Although Trop-2 expression was identified as a favorable prognostic factor, its broad expression suggests that sacituzumab govitec may be a potential therapeutic option in a wide spectrum of patients with UTUC. Our future task is to evaluate the actual response rate of sacituzumab govitecan for UTUC in clinical practice and to assess its relationship with Trop-2 expression patterns.

## Figures and Tables

**Table 1:** Clinicopathologic characteristics and outcome of patients with upper tract urothelial carcinoma.

Variable	Immunohistochemical Analysis	RNA Sequencing Analysis
	*n* = 99	*n* = 72
Age (year), median (range)	71 (48–87)	73 (45–89)
Sex, *n* (%)		
Male	60 (60.6)	55 (76.4)
Female	39 (39.4)	17 (23.6)
Laterality, *n* (%)		
Right	43 (43.4)	38 (52.8)
Left	56 (56.6)	34 (47.2)
Tumor location, *n* (%)		
Renal pelvis	45 (45.5)	27 (37.5)
Ureter	50 (50.5)	43 (59.7)
Both	4 (4.0)	2 (2.8)
Tumor grade, *n* (%)		
Low-grade	15 (15.2)	8 (11.1)
High-grade	84 (85.9)	64 (88.9)
Pathological T stage, *n* (%)		
pTa	19 (19.2)	14 (19.4)
pT1	18 (18.2)	22 (30.6)
pT2	8 (8.1)	10 (13.9)
pT3	48 (48.5)	23 (31.9)
pT4	6 (6.1)	3 (4.2)
Lymphovascular invasion, *n* (%)		
Yes	40 (40.4)	50 (69.4)
No	59 (59.6)	22 (30.6)
Lymph node metastasis, *n* (%)		
pN0	84 (84.8)	63 (87.5)
pN+	12 (12.1)	9 (12.5)
pNx	3 (3.0)	0 (0.0)
High-risk group ^†^, *n* (%)		
Yes	60 (60.6)	33 (45.8)
No	36 (36.4)	39 (54.2)
Unknown	3 (3.0)	0 (0.0)
Adjuvant chemotherapy, *n* (%)		
Yes	26 (26.3)	12 (16.7)
No	63 (63.6)	60 (83.3)
Progression, *n* (%)		
Yes	38 (38.4)	25 (34.7)
No	61 (61.6)	47 (65.2)
Follow-up (month), median (range)	37 (1–173)	28 (2–88)

^†^ High-risk group is defined as patients with a pathologic stage of ≥pT3 or positive lymphatic invasion or lymph node metastasis.

**Table 2:** Association of Trop-2 expression with clinicopathological characteristics of upper tract urothelial carcinoma.

Variable	*n*	Trop-2 expression				*p*		
		0 + (%)	1 + (%)	2 + (%)	3 + (%)	0 vs. 1 +/2 +/3 +	0/1 + vs. 2 +/3 +	0/1 +/2 + vs. 3 +
Sex						0.077	0.37	0.82
Male	60	1 (1.7)	15 (25.0)	26 (43.3)	18 (30.0)			
Female	39	4 (10.3)	10 (25.6)	16 (41.0)	9 (23.1)			
Tumor site						0.37	0.66 ^a^	0.82 ^a^
Renal pelvis	45	1 (2.2)	12 (26.7)	20 (44.4)	12 (26.7)			
Ureter	50	4 (8.0)	13 (26.0)	20 (40.0)	13 (26.0)			
Both	4	0 (0.0)	0 (0.0)	2 (50.0)	2 (50.0)			
Tumor grade						0.57	0.54	1.00
Low-grade	15	1 (6.7)	2 (13.3)	8 (53.3)	4 (26.7)			
High-grade	84	4 (4.8)	23 (27.4)	34 (40.5)	23 (27.4)			
Pathological stage						0.65 ^b^	1.00 ^b^	0.020 ^b^ **
pTa	19	1 (5.3)	5 (26.3)	4 (21.1)	9 (47.4)			
pT1	18	0 (0.0)	5 (27.8)	7 (38.9)	6 (33.3)			
pT2	8	0 (0.0)	2 (25.0)	4 (50.0)	2 (25.0)			
pT3	48	3 (6.3)	12 (25.0)	24 (50.0)	9 (18.8)			
pT4	6	1 (16.7)	1 (16.7)	3 (50.0)	1 (16.7)			
Lymphovascular invasion						0.39	1.00	0.069
No	59	2 (3.4)	16 (27.1)	21 (35.6)	20 (33.9)			
Yes	40	3 (7.5)	9 (22.5)	21 (52.5)	7 (17.5)			
Lymph node involvement						0.075 ^c^	0.74	0.50 ^c^
pN0	84	2 (2.4)	22 (26.2)	35 (41.7)	25 (29.8)			
pN+	12	2 (16.7)	2 (16.7)	6 (50.0)	2 (16.7)			
pNx	3	1 (33.3)	1 (33.3)	1 (33.3)	0 (0.0)			

^a^ Renal pelvis vs. ureter; ^b^ pTa + pT1 vs. pT2 + pT3 + pT4; ^c^ pN0 vs. pN+; ** *p* < 0.05.

**Table 3:** Univariate and multivariate analyses of progression-free survival and cancer-specific survival in patients with upper tract urothelial carcinoma.

	Progression-Free Survival						Cancer-Specific Survival					
Variable	Univariate			Multivariate			Univariate			Multivariate		
	HR	95 %CI	*p*	HR	95 %CI	*p*	HR	95 %CI	*p*	HR	95 %CI	*p*
All cases (*n* = 99)												
Sex (male/female)	1.33	0.68–2.61	0.40				1.33	0.62–2.85	0.46			
Age (70</≤70)	1.38	0.72–2.64	0.33				1.65	0.78–3.48	0.19			
Tumor site (pelvis/ureter)	0.80	0.41–1.55	0.50				0.83	0.40–1.72	0.61			
Tumor grade (high/low)	4.44	1.07–18.51	0.041 **	5.52	1.29–23.72	0.022 **	7.77	1.06–57.18	0.044 **	6.95	0.94–51.23	0.057 *
pT stage (MI/NMI)	17.30	4.15–72.14	<0.001 **	8.5	1.94–37.31	0.0046 **	1.09 × 10^9^	13.52–Inf	<0.001 **	5.76 × 10^9^	(6.39–7.60) × 10^38^	<0.001 **
Lymphovascular invasion (Yes/No)	5.84	2.88–11.83	<0.001 **	2.54	1.10–5.84	0.028 **	5.62	2.50–12.66	<0.001 **	1.86	0.77–4.48	0.16
Lymph node metastasis (Yes/No)	4.40	2.07–9.36	<0.001 **	2.06	0.95–4.47	0.068 *	2.74	1.17–6.40	0.020 **	1.07	0.43–2.63	0.89
Adjuvant chemotherapy (Yes/No)	1.13	0.56–2.28	0.73				1.08	0.49–2.37	0.85			
Trop-2 strong expression	0.18	0.055–0.58	0.0043 **	0.29	0.087–0.97	0.045 **	0.16	0.039–0.69	0.014 **	0.31	0.072–1.36	0.12

Abbreviations: CI, confidence interval; HR, hazard ratio; Inf, Infinity; MI, muscle invasive; NMI, non–muscle invasive; ** *p* < 0.05 and * *p* < 0.1.

**Table 4:** Univariate and multivariate analyses of progression-free survival and cancer-specific survival in patients with high-risk upper tract urothelial carcinoma.

	Progression-Free Survival						Cancer-Specific Survival					
Variable	Univariate			Multivariate			Univariate			Multivariate		
	HR	95 %CI	*p*	HR	95 %CI	*p*	HR	95 %CI	*p*	HR	95 %CI	*p*
High-risk group ^†^ (*n* = 60)												
Sex (male/female)	1.35	0.67–2.72	0.40				1.23	0.58–2.65	0.6			
Age (70</≤70) years	1.11	0.56–2.18	0.77				1.57	0.73–3.37	0.24			
Tumor site (pelvis/ureter)	0.95	0.48–1.91	0.89				0.97	0.46–2.04	0.93			
Tumor grade (high/low)	6.96	0.95–51.06	0.056 *	7.91	1.07–58.25	0.042 **	6.41	0.87–47.47	0.069 *	7.09	0.96–52.49	0.055 *
Adjuvant chemotherapy (Yes/No)	0.71	0.35–1.46	0.36				0.63	0.28–1.41	0.26			
Trop-2 strong expression	0.30	0.092–0.99	0.048 **	0.268	0.081–0.88	0.031 **	0.29	0.069–1.23	0.093 *	0.26	0.061–1.10	0.067 *

^†^ High-risk group is defined as patients with a pathologic stage of ≥pT3 or positive lymphatic invasion or lymph node metastasis; ** *p* < 0.05 and * *p* < 0.1. Abbreviations: CI, confidence interval; HR, hazard ratio.

## Data Availability

The data presented in this study are available on request from the corresponding author.

## Supplementary Materials

The following supporting information can be downloaded at: https://www.mdpi.com/article/10.3390/curroncol29060312/s1. Table S1: Univariate and multivariate analyses of progression-free survival in patients with upper tract urothelial carcinoma in RNA sequencing analysis, Figure S1: Progression-free survival (A) and cancer-specific survival (B) in 62 patients with muscle-invasive upper tract urothelial carcinoma stratified by the expression of Trop-2 (0/1+/2+ vs. 3+).
