# Supplementary material for: Trop-2 in Upper Tract Urothelial Carcinoma
Source: Curr Oncol. 2022 May 30;29(6):3911–21. doi: 10.3390/curroncol29060312 (PMC9222112; doi:10.3390/curroncol29060312)
Supplement: Supplementary file 1 [file curroncol-29-00312-s001.zip › curroncol-1720213-supplementary.pdf]

Current Oncology

Table S1. Univariate and multivariate analyses of progression-free survival in patients with upper tract urothelial carcinoma in RNA sequencing analysis.

Variable	Progression-free survival					
	Univariate			Multivariate		
	HR	95 %CI	p	HR	95 %CI	p
All cases (n = 72)						
Sex (male/female)	0.58	0.25–1.34	0.20			
Age (70 < / ≤ 70) years	1.13	0.50–2.57	0.77			
Tumor site (pelvis/ureter)	0.80	0.34–1.88	0.61			
Tumor grade (high/low)	1.93	0.45–8.23	0.38			
pT stage (MI/NMI)	4.13	1.64–10.38	0.0026**	2.47	0.14–1.15	0.090*
Lymphovascular invasion (Yes/No)	3.52	1.59–7.80	0.0019**	1.86	0.72–4.77	0.20
Lymph node involvement (Yes/No)	3.35	1.31–8.57	0.012**	1.56	0.55–4.46	0.40
Adjuvant chemotherapy (Yes/No)	2.45	1.05–5.68	0.038**	1.68	0.61–4.64	0.32
TACSTD2 expression (high/low)	0.47	0.21–1.06	0.069*	1.92	0.78–4.74	0.16
High-risk group† (n = 33)						
Sex (male/female)	0.71	0.26–1.93	0.51	0.51	0.14–1.81	0.30
Age (70 < / ≤ 70)	0.92	0.34–2.46	0.86	0.70	0.15–3.17	0.64
Tumor site (pelvis/ureter)	0.78	0.28–2.15	0.63	0.80	0.20–3.21	0.76
Adjuvant chemoterapy (Yes/No)	0.98	0.35–2.74	0.97	1.36	0.33–5.67	0.67
TACSTD2 expression (high/low)	0.33	0.11–0.95	0.039**	0.21	0.057–0.75	0.017**

Abbreviations: CI, confidence interval; HR, hazard ratio; MI, muscle invasive; NMI, non–muscle invasive.

† High-risk group is defined as patients with a pathologic stage of ≥ pT3 or positive lymphatic invasion or lymph node metastasis; **p < 0.05 and *p < 0.1.

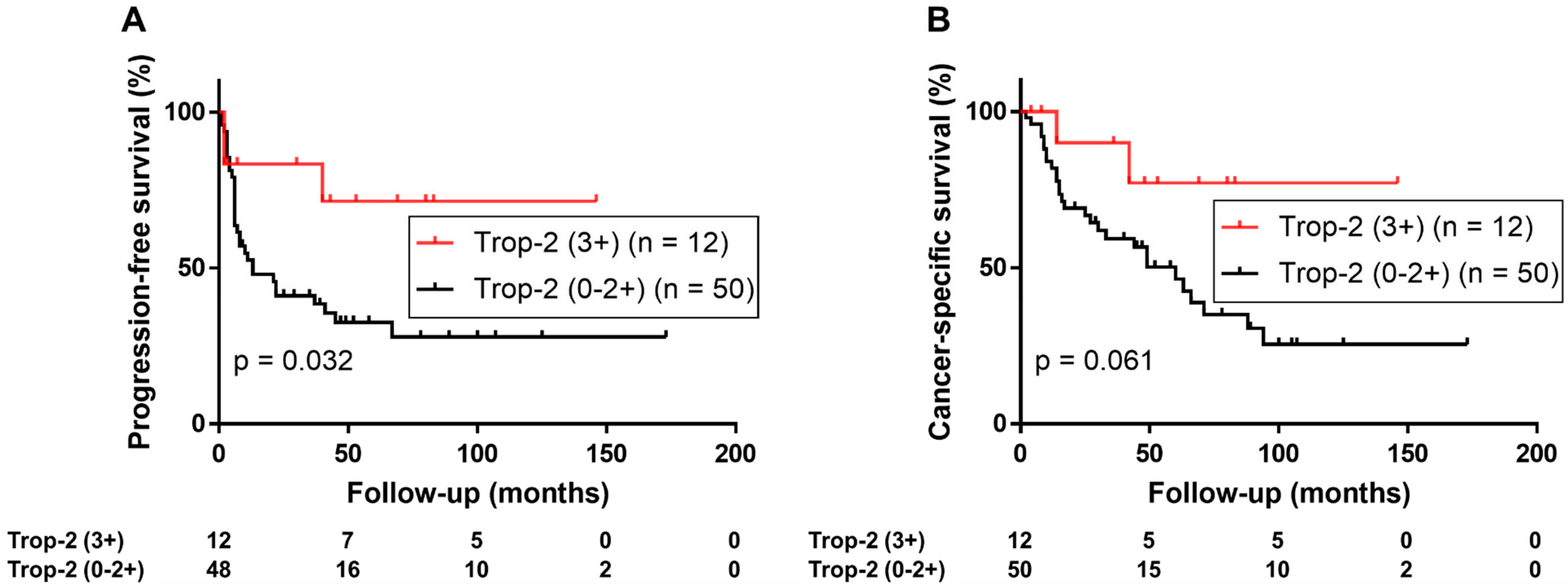

Figure S1. Progression-free survival (**A**) and cancer-specific survival (**B**) in 62 patients with muscle-invasive upper tract urothelial carcinoma stratified by the expression of Trop-2 (0/1+/2+ vs 3+).
